# Biodegradable microspheres via orally deliver celastrol with ameliorated neuropathic pain in diabetes rats

**DOI:** 10.1093/rb/rbae087

**Published:** 2024-07-17

**Authors:** Haosen Zhao, Shurui Chen, Sen Lin, Xifan Mei

**Affiliations:** Department of Orthopaedic Rehabilitation, Third Affiliated Hospital of Jinzhou Medical University, Jinzhou, Liaoning Province, 121000, P. R. China; Cardiac Intensive Care Unit, Cardiovascular Hospital, Guangdong Second Provincial General Hospital, Guangzhou, Guangdong Province, 510317, P. R. China; Department of Orthopaedic Rehabilitation, Third Affiliated Hospital of Jinzhou Medical University, Jinzhou, Liaoning Province, 121000, P. R. China; Department of Orthopaedic Rehabilitation, Third Affiliated Hospital of Jinzhou Medical University, Jinzhou, Liaoning Province, 121000, P. R. China

**Keywords:** biodegradable spheres, neuropathic pain, diabetes, immunoengineering, reactive oxygen species

## Abstract

The treatment of peripheral neuropathy resulting from diabetes primarily emphasizes neurotrophic medications. However, a growing body of clinical studies indicates that neuroinflammation plays a significant role in the pathogenesis of neuropathic pain. This has spurred active exploration of treatment strategies leveraging nanomedicine for diseases, aiming for superior therapeutic outcomes. In this context, we have developed biodegradable nanoparticles made of polylactic-co-glycolic acid, loaded with triptolide (pCel), designed to alleviate somatic cell neuropathic pain induced by diabetes. Treatment with pCel notably reduced levels of reactive oxygen species and apoptosis *in vitro*. Furthermore, the progression of streptozotocin-induced diabetes, characterized by elevated renal function indices (blood urea nitrogen, creatinine), liver function indices (bilirubin, alkaline phosphatase) and decreased levels of albumin and globulin, was mitigated following pCel administration. Importantly, oral treatment with pCel significantly inhibited mechanical allodynia and the activation of the sciatic glial cells in diabetic rats. These findings indicate that this synthetic, biodegradable nanomedicine exhibits excellent stability, biocompatibility and catalytic activity, making it a promising and innovative approach for the management of chronic pain conditions associated with diabetic neuropathy.

## Graphical Abstract

**Figure:**
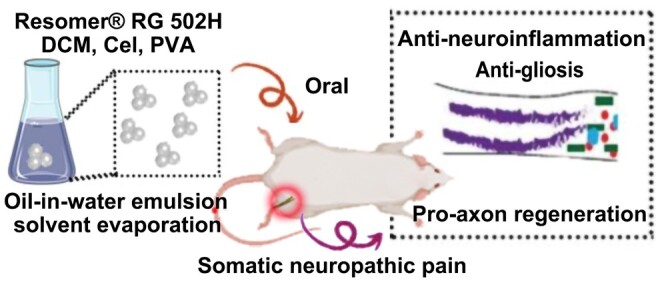

## Introduction

Chronic hyperglycemia and impaired glucose homeostasis are hallmark characteristics of diabetes mellitus. With the aging population, the prevalence of diabetes continues to rise. According to the latest data from 2021, an estimated 536.6 million people worldwide have diabetes (a prevalence rate of 10.5%), up from the 2019 estimate of 463.9 million (a prevalence rate of 9.3%) [1, 2]. Over time, these issues can precipitate secondary complications that pose significant health risks, potentially becoming life-threatening [3]. Effective diabetes management requires a multifaceted strategy. An integrated treatment approach is essential, incorporating lifestyle modifications alongside a combination therapy regimen. This regimen typically includes anti-hyperglycemic agents, anti-hypertensive medications and antihyperlipidemic drugs [4, 5]. In cases where diabetes remains inadequately managed, there is a progressive decline in β-cell function due to either the failure of insulin secretion or the dysregulation of neuroendocrine signaling pathways. Such a decline often leaves patients with no alternative but to rely on insulin supplementation for glycemic control [6–8]. Although new glucose-responsive insulin delivery systems have been developed in recent years, how to more effectively control blood sugar and prevent or treat complications of diabetes remains a key issue in current research [9].

One of the most prevalent complications arising from diabetes is a collection of clinical syndromes linked to damage within the peripheral and autonomic nervous systems [10]. Up to half of the individuals diagnosed with diabetes may experience these syndromes, collectively known as various forms of neuropathy, stemming from both diffuse and focal neurological damage [11, 12]. Both type 1 diabetes and type 2 diabetes patients are prone to developing secondary diabetic complications. Diabetic peripheral neuropathy is the most common complication, characterized by length-dependent damage to peripheral nerves, starting in the feet and progressing proximally. This condition severely affects patients, causing pain, loss of limb sensation, falls and foot ulcers. In the most severe cases, it can even lead to amputation or death. Throughout this study, we will specifically focus on diabetic neuropathy, particularly emphasizing neuropathic pain, as the most frequently encountered form of neuropathy. Notably, an upsurge in the activity of inflammation-related pathways has been observed in sciatic nerve arrays within preclinical models of diabetes. Given the limitations of existing treatments for the primary disease, there is a significant interest in exploring the potential of targeting inflammation more directly. This approach aims to enhance both the prevention and management of diabetic neuropathy [13].

Recent studies, both *in vitro* and *in vivo*, underscore the potential benefits of natural anti-inflammatory agents in addressing complications associated with diabetes [14–18]. Celastrol (Cel), in particular, has emerged as a compound of significant interest due to its efficacy across a broad spectrum of metabolic disorders [19]. Its anti-obesity effects, conclusively identified by the Ozcan group [20], have propelled Cel into the spotlight for its potential in metabolic disease management. However, the transition of Cel from research to clinical application faces a major hurdle: its low oral bioavailability, attributed to extensive systemic metabolism, presents a considerable challenge for its clinical translation.

Thus, in this study, we aim to develop a novel approach for Cel-controlled and sustained release as a new alternative therapeutic method with improved safety and oral bioavailability. In this work, we describe the design and development of Cel-loaded biodegradable poly (lactic-co-glycolic acid) nanoparticles by the oil-in-water emulsion/evaporation technique, besides the physicochemical characterization. Using the STZ-induced diabetic rodent model, we analyzed the role of pCel to determine how it affects the progression of neuropathic pain.

## Materials and methods

### Materials

All chemicals were purchased from Sigma-Aldrich unless otherwise specified. All antibodies were obtained from Abcam unless otherwise specified. Interleukin-1 beta (IL-1β) and tumor necrosis factor-alpha ELISA kits were obtained from Absin. Reactive oxygen species (ROS) staining kits were purchased from Solarbio.

### Animals

All animal experiments were performed in strict accordance with the guidelines for the care and use of laboratory animals and received approval from the Institutional Biomedical Research Ethics Committee of Jinzhou Medical University. The ethical approval number for the experimental animals is 2022007. Male Sprague Dawley rats, weighing between 180 and 220 g and aged 12 months, were housed under specific pathogen-free conditions within the facilities of the Animal Care Unit at Jinzhou Medical University. The animals were provided with *ad libitum* access to food and water, ensuring their well-being throughout the duration of the study.

### Preparation and characterization of pCel

The preparation of pCel involved the initial preparation of the oil and aqueous phases. The oil phase consisted of dichloromethane (DCM) (5 ml), Cel (50 mg), and Resomer RG 502H (Mw: 17 000 Da; lactide: glycolide ratio = 50:50) (400 mg). The aqueous phase comprised a 1% (w/v) polyvinyl alcohol (PVA) solution (15 ml). Additionally, a maturation medium of 0.1% (w/v) PVA aqueous solution (50 ml) was prepared.

The oil phase was created by dissolving Cel and the polymer in DCM, utilizing vortex stirring (Vortex Genius 3, VWR International, Germany) until fully dissolved. Subsequently, the aqueous phase was gradually introduced to the oil phase, followed by mechanical homogenization (Ultra-Turrax T25, IKA Laboratories, Staufen, Germany) to form the oil-in-water emulsion. This process was conducted at 8000 rpm for 30 s within an ice bath to prevent DCM evaporation.

The emulsion was then transferred to the maturation medium and incubated for 3 h at 40°C with magnetic stirring at 100 rpm to facilitate the evaporation of the organic solvent. The pCel nanoparticles were isolated using sequential filtration through nylon sieves of 25 µm and then 10 µm to achieve the desired size range. The nanoparticles were washed three times, filtered and vacuum-dried over a 24-h period.

Characterization of the pCel involved determining particle size and size distribution via dynamic light scattering. The morphological structures of pCel were examined using transmission electron microscopy (JEOL-2100).

### STZ-induced diabetes and pCel administration

Diabetes was induced in male Sprague Dawley rats through a single intraperitoneal injection of STZ at a dose of 55 mg/kg, dissolved in a 0.1 M citrate buffer at a pH of 4.5. Following a 72-h period, during which the last 16 h included fasting, blood glucose levels were measured. Rats exhibiting glucose levels exceeding 150 mg/dl were deemed diabetic and selected for inclusion in this 10-week longitudinal study. These animals were subsequently randomized into three distinct groups for the investigation: Control (*n* = 8, healthy non-diabetic rats), STZ (*n* = 8, diabetic rats without treatment) and STZ + pCel (*n* = 8; administered 20 mg/kg/day of pCel orally).

A cohort of 24 rats was subjected to STZ treatment. By the conclusion of the study, all rats had survived without encountering any complications aside from the manifestations of diabetes, affirming the efficacy and safety of the experimental procedures employed.

### Blood collection and tissue sampling procedure

The animals were fasted overnight for approximately 16 h on the last day of the experimental model before the induction of anesthesia or the collection of blood samples. Throughout the study, blood samples were periodically collected from the rats via tail prick every other week for the purpose of glucose measurement. This was accomplished using a glucometer, and the procedure was repeated until the conclusion of the study period. At the time of sacrifice, blood was obtained through cardiac puncture and subsequently centrifuged at 3000 g for 20 min to separate serum/plasma. These samples were then preserved at 4°C for future analyses. Additionally, muscle and nerve tissues were aseptically excised and either stored at −80°C or fixed in formalin, pending further examination.

### Tissue processing and immunohistochemistry

Muscle and nerve tissue samples were first prepared by freezing, followed by embedding in Optimum Cutting Temperature compound (Sakura Finetek, Leiden, The Netherlands) to facilitate cryostat sectioning at −20°C. Tissue sections were cut to a uniform thickness of approximately 5 μm for subsequent immunohistochemical analysis.

To permeabilize the cells, tissue sections were incubated with 0.3% Triton X-100 in phosphate-buffered saline (PBS) for 30 min, followed by a dual PBS wash to remove any residual detergent. The sections were then blocked with 1% bovine serum albumin in PBS for 45 min to prevent non-specific antibody binding. For the immunodetection process, the sections were incubated with specific primary antibodies overnight at 4°C. This step was succeeded by a 12-h incubation with appropriate secondary antibodies at 4°C, facilitating the visualization of the target proteins.

### Assessment of mechanical allodynia and thermal hyperalgesia

Mechanical pain thresholds were quantitatively assessed at various time points before and after treatment administration. Mechanical allodynia was evaluated by determining the foot withdrawal threshold in response to a mechanical stimulus applied to each hind paw. This stimulus was delivered using a sharp, cylindrical probe with a uniform tip diameter of 0.2 mm, attached to an Electro von Frey apparatus. The probe was precisely applied to six designated loci on the plantar surface of the foot. The minimal force (in grams) required to induce paw withdrawal was digitally recorded. The threshold for mechanical withdrawal for each animal was established by averaging these six readings, with the force subsequently converted into milli-Newtons. The reported results represent mean values for the ipsilateral feet.

Thermal hyperalgesia was assessed by measuring the latency of foot withdrawal in response to heat stimulation. A heat source provided by an analgesia meter was employed for this purpose. Each animal was positioned within an enclosure featuring a smooth, temperature-regulated glass floor. The heat source was precisely targeted at a specific area of the hind paw, which was in direct contact with the glass surface, and a radiant thermal stimulus was applied to this region. The stimulus was automatically terminated upon movement of the hind paw (or after a maximum duration of 30 s to prevent potential tissue damage). The intensity of the heat stimulus was consistently controlled across all experimental sessions. Normally, control animals displayed paw movement at latencies ranging from 5 to 10 s. Thermal stimuli were administered thrice to each hind paw with 5–6 min intervals between applications. For expressing results related to mechanical allodynia or thermal hyperalgesia, mean values for ipsilateral feet are provided. To ensure the integrity of the behavioral assessments, experimenters conducting both mechanical and thermal tests were blinded to the treatment conditions of the subjects.

### RT-qPCR analysis

Tissue samples were harvested specifically for the purpose of RT-qPCR analysis to quantify the relative expression levels of target genes. These expression levels were normalized against the housekeeping gene, β-actin, to ensure accuracy in quantification. For comparative analysis, the relative expression of target genes in the experimental group was assessed against that of the control group. This comparison was facilitated using the 2 ^ (−ΔΔCT) method, a widely recognized approach for calculating fold changes in gene expression from qPCR experiments.

### Statistical analysis

The data collected in this study are presented as mean ± standard deviation to convey central tendency and variability. For the evaluation of differences among multiple groups, a one-way analysis of variance was utilized. This statistical approach facilitated the identification of any significant disparities across the experimental conditions examined. All analyses and graphing were conducted using GraphPad Prism software (version 9.4.0, San Diego CA, USA), ensuring rigorous and reproducible results.

## Results and discussion

### Preparation and characterization of pCel

Initially, the constituent phases were meticulously prepared. The oil phase was constituted of DCM (5 ml), Cel (50 mg), and Resomer RG 502H (Mw: 17 000 Da; lactide: glycolide ratio = 50:50) (400 mg). Concurrently, the aqueous phase was formulated from a 1% w/v PVA solution (15 ml). Additionally, a maturation medium comprising a 0.1% w/v PVA aqueous solution (50 ml) was also prepared.

The internal phase was concocted by dissolving both the polymer and drug into the organic solvent via vortex stirring (Vortex Genius 3, VWR International), continued until full dissolution was observed. Following this, an oil-in-water emulsion was generated through mechanical homogenization, which involved the gradual integration of the aqueous phase into the organic phase at 8000 rpm for 30 s.

**Figure 1.**
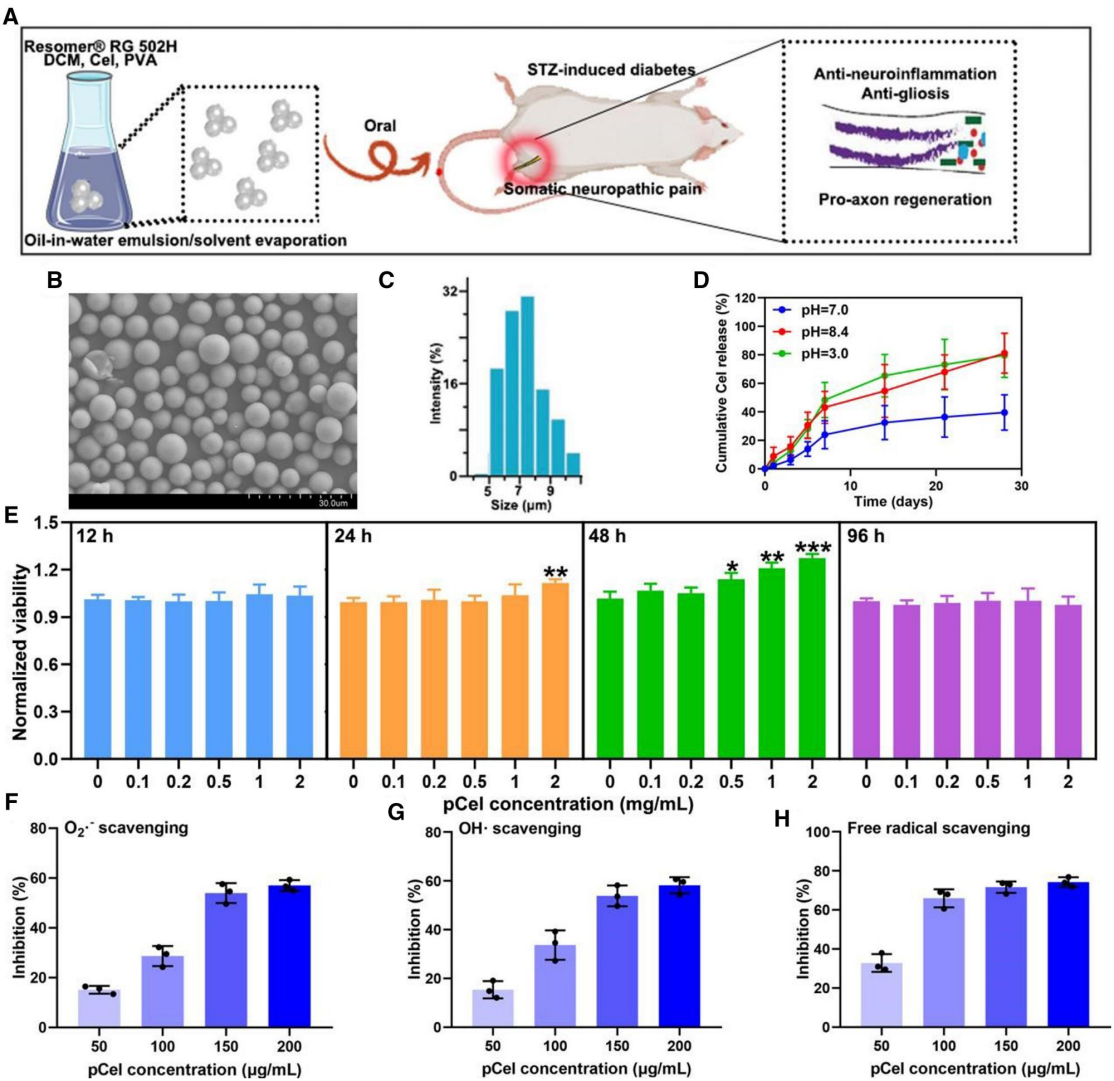
Characterization of the pCel. (**A**) Schematic illustration of pCel synthesis and NPs-based treatment for STZ-induced somatic neuropathic pain *in vivo*. (**B**) Scanning electron microscope images of pCel. (**C**) Dynamic light scattering of Cel. (**D**) Cumulative Cel release analysis of pCel. (**E**) Neuronal viabilities test of pCel. (**F**) O^2−^ scavenging activities of Cel. (**G**) ·OH scavenging activities of Cel. (**H**) Free radical scavenging activities of Cel. *P < 0.05, **P < 0.01, ***P < 0.001.

Subsequently, this emulsion was introduced into the maturation medium and maintained at 40°C with magnetic stirring at 100 rpm, to expedite the evaporation of the organic solvent. To achieve the nanoparticles in the desired size range, the pCel were sequentially filtered through a 25-µm nylon sieve and then a 10-µm nylon filter. The resultant pCel nanoparticles underwent three cycles of washing, filtering and vacuum-drying to ensure purity and stability (Figure 1A).

Scanning electron microscopy imaging, as depicted in Figure 1B, revealed that the synthesized pCel microspheres are uniformly spherical, indicative of robust structural integrity. This uniformity suggests a controlled and consistent preparation process. The particle size analysis, detailed in Figure 1C, showed an average diameter of approximately 7.2 µm for the pCel particles.

The release dynamics of Cel from the pCel microspheres were rigorously evaluated under different pH conditions to mimic the gastrointestinal environment (Figure 1D). Notably, at pH 7.0, simulating the acidic gastric environment, approximately 36% of Cel was released within 24 h. In contrast, the release rate significantly increased to approximately 80% in a pH 8.4 and pH 3.0 setting, akin to the intestinal and gastric microenvironment, respectively. But with the administration of pCel the gastric emptying has not increased (1–2 h). This enhanced release at neutral pH can be attributed to the accelerated dissociation of Cel in the intestinal milieu, highlighting the potential efficacy of pCel for oral delivery in navigating the varied pH landscapes of the gastrointestinal tract.

Diabetic neuropathy, which is characterized by axonal damage to sensory, autonomic and eventually motor axons, represents a distinct neurodegenerative disorder of the peripheral nervous system [21]. Given the critical role of neurons in mediating somatic neuropathic pain, assessing the cytocompatibility of pCel is paramount. Our evaluation focused on the impact of pCel on the activity of primary neurons’ dehydrogenases, as depicted in Figure 1E. Within the concentration range of 0.1–2.0 mg/ml and over a testing period of 12–96 h, pCel exhibited no cytotoxic effects. Remarkably, neuronal activity was enhanced following treatment with pCel for both 24 and 48 h. A significant increase in neuron viability was noted at the highest tested concentration of pCel (2.0 mg/ml) after 24 h of treatment. This improvement in viability persisted with a concentration of 0.5 mg/ml even when the treatment duration was extended to 48 h. However, no enhancement in neuronal activity was observed at 96 h, potentially due to the accumulation of cellular metabolic byproducts in the medium. These findings underscore the neuroprotective potential of pCel, particularly effective within the concentration range of 0.1–2.0 mg/ml, in mitigating the deleterious effects associated with diabetic neuropathy.

Oxidative stress plays a significant role in the process of tissue damage. It occurs when there is an imbalance between the production of ROS and the body’s ability to neutralize or repair their harmful effects. This imbalance can lead to cellular and molecular damage, contributing to various pathological conditions. Recent studies have highlighted the importance of ROS in the development and progression of tissue damage, emphasizing the need for effective antioxidant strategies to mitigate these effects [22–24]. To elucidate the antioxidative efficacy of pCel, its capacity to scavenge ROS, specifically superoxide anion (O^2−^) and hydroxyl radical (·OH), was investigated. The ROS scavenging activity of pCel was found to be concentration-dependent, as demonstrated in Figure 1F and G. At a concentration of 150 µg/ml, pCel was able to decompose approximately 53% of the total O^2−^, as illustrated in Figure 1F. Similarly, treatment with 150 µg/ml pCel resulted in the decomposition of about 60% of ·OH radicals, as depicted in Figure 1G.

Further confirmation of pCel’s antioxidative properties was obtained through the 2,2′-azino-bis(3-ethylbenzothiazoline-6-sulfonate) (ABTS) radical scavenging assay. This assay, designed to quantify the scavenging of free radicals, revealed that pCel at a relatively low concentration (150 mg/l) could eliminate more than 75% of the ABTS radicals, as shown in Figure 1H.

### Anti-ROS and anti-apoptosis effects of pCel

**Figure 2.**
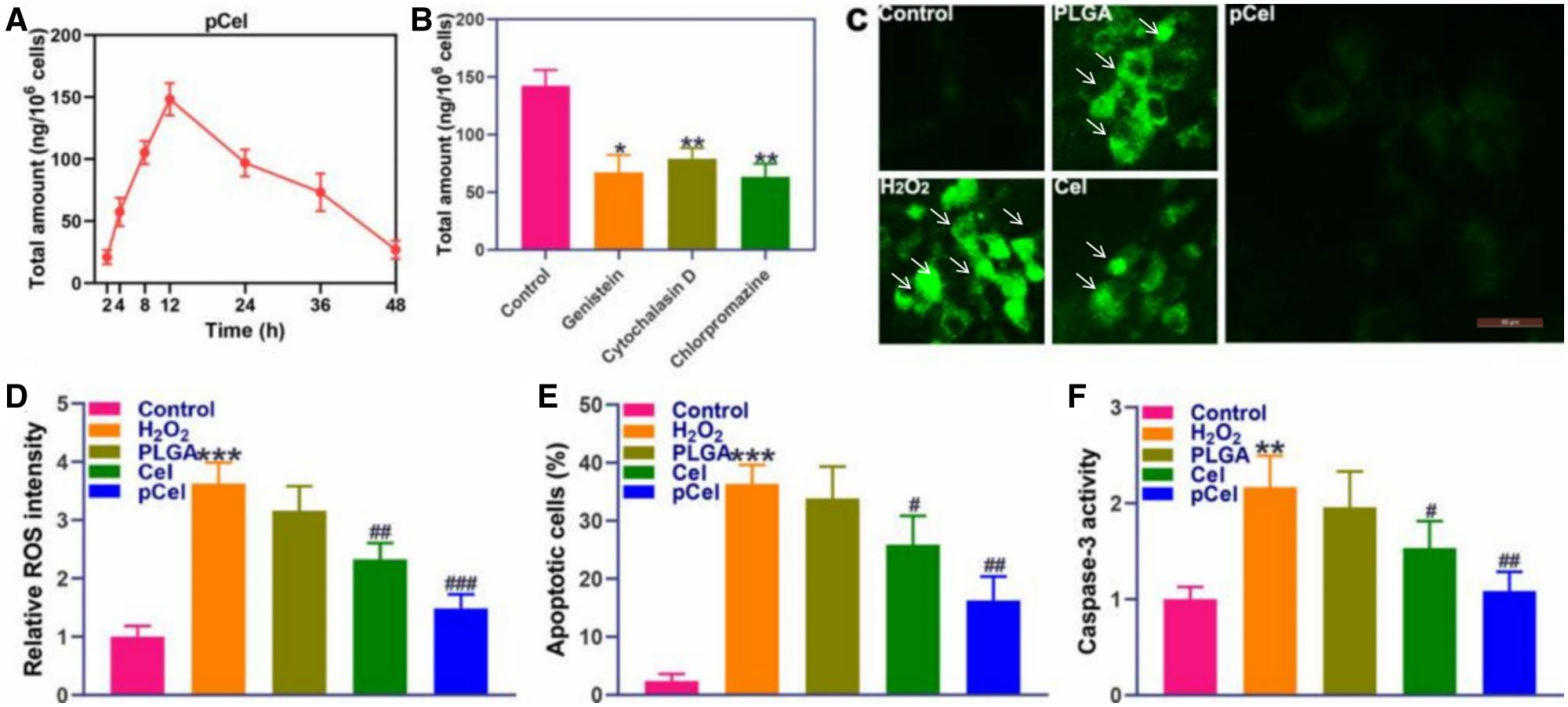
Anti-ROS and anti-apoptosis effects of the pCel. (**A**) The content of pCel in primary neurons. (**B**) The content of pCel in primary neurons treated with various endocytosis inhibitors. (**C**) ROS staining of pCel in primary neurons (400×). (**D**) Representative ROS quantification of pCel in primary neurons. (**E**) Representative apoptotic quantification of pCel in primary neurons. (**F**) Representative caspase-3 quantification of pCel in primary neurons. **P < 0.01, ***P < 0.001, compared to the control group. #*P* < 0.05, ##*P* < 0.01, ###*P* < 0.001, compared to H_2_O_2_ group.

Cellular uptake of pCel by primary neurons was quantitatively assessed using inductively coupled plasma mass spectrometry, showing maximal accumulation after 12 h of incubation, indicative of effective internalization (Figure 2A). To investigate the uptake mechanism, primary neurons were co-treated with endocytosis inhibitors (genistein, cytochalasin D, and chlorpromazine), with cytochalasin D and chlorpromazine significantly reducing the cellular uptake of pCel nanoparticles, suggesting endocytosis as the primary internalization pathway (Figure 2B). Intracellular ROS production was examined following 200 mM H_2_O_2_ treatment for 30 min, which significantly increased intracellular ROS levels and altered cell morphology, resulting in abnormal and shrinking cell anatomy; however, these effects were mitigated by pCel treatment for 12 h, as evidenced by reduced intracellular ROS levels confirmed by flow cytometry (Figure 2C and D). Among the caspases found, caspase-3 is responsible for executing apoptosis, cleaving a number of important substrates downstream [25]. Further analysis in apoptosis and caspase-3 activity revealed that pCel substantially reduced H_2_O_2_-induced apoptosis, as shown by flow cytometry (Figure 2E), and inhibited the upregulation of caspase-3 expression in H_2_O_2_-exposed neurons, underscoring pCel's ROS scavenging and cytoprotective effects at the cellular level (Figure 2F).

### Prevention of STZ-induced diabetes and mechanical allodynia via pCel treatment *in vivo*

**Figure 3.**
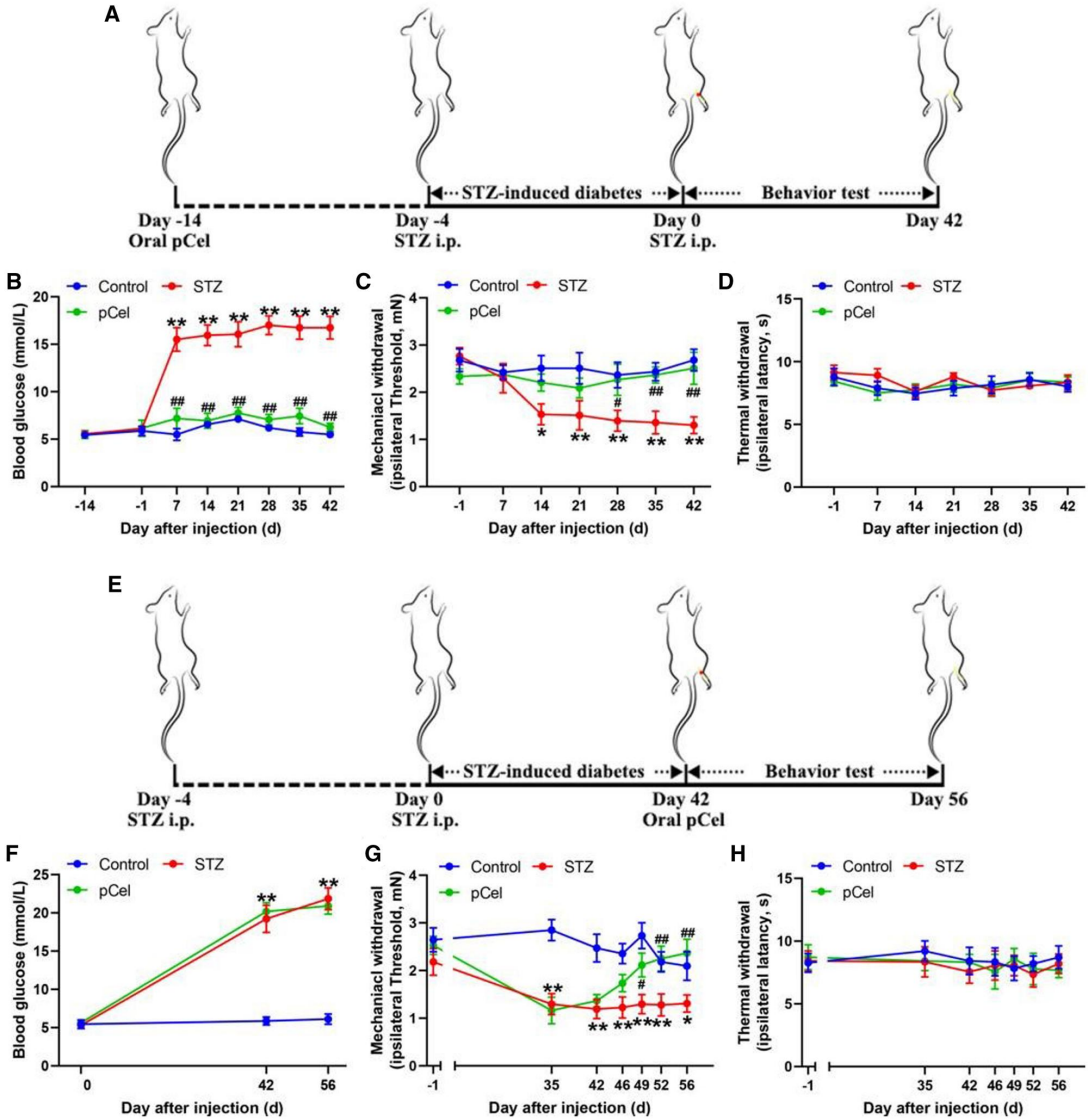
Prevention of STZ-induced diabetes and mechanical allodynia via pCel treatment *in vivo*. (**A**) Continuous feeding of pCel begins 2 weeks before STZ treatment. (**B**) Representative quantification of blood glucose in STZ-induced diabetes. (**C**) Representative quantification of mechanical withdrawal in STZ-induced diabetes. (**D**) Representative quantification of thermal withdrawal in STZ-induced diabetes. (**E**) Continuous feeding of pCel begins 7 weeks later than STZ treatment. (**F**) Representative quantification of blood glucose in STZ-induced diabetes. (**G**) Representative quantification of mechanical withdrawal in STZ-induced diabetes. (**H**) Representative quantification of thermal withdrawal in STZ-induced diabetes. *P < 0.05, **P < 0.01, compared to the control group. #*P* < 0.05, ##*P* < 0.01, compared to STZ group.

Diabetic neuropathic pain, a prevalent form of neuropathic discomfort, was investigated in this study to determine the role of pCel in its development *in vivo* [10]. STZ administration, which selectively destroys pancreatic islet β-cells leading to insulin deficiency and hyperglycemia, mimics type 1 diabetes mellitus in humans. Following repeated STZ injections (intraperitoneally, 40 mg/kg, once daily for five consecutive days) as depicted in Figure 3A, significant and persistent increases in blood glucose levels were observed (Figure 3B), inducing mechanical allodynia without affecting thermal sensitivity (Figure 3C and D). Remarkably, pretreatment with pCel for two weeks prior to STZ administration effectively prevented the STZ-induced hyperglycemia (Figure 3B) and the subsequent development of mechanical allodynia and thermal hypersensitivity, indicating the absence of diabetic conditions in these rats (Figure 3C and D).

To more closely mimic the typical clinical treatment scenario, pCel administration was initiated 42 days following STZ treatment, as shown in Figure 3E. Blood samples from all rats were analyzed for biomarkers including blood urea nitrogen, creatinine, albumin, globulin, bilirubin and alkaline phosphatase, to assess liver and kidney functions. In diabetic rats, these biomarkers deviated significantly from the normal ranges, indicating impaired liver and kidney functions, as detailed in Supplementary Figures S1–S6. Remarkably, treatment with pCel normalized these biomarkers. Although pCel did not influence the established hyperglycemia (Figure 3F), it significantly mitigated mechanical allodynia (Figure 3G) while having no effect on thermal sensitivity (Figure 3H).

Hematological assays indicated reduced concentrations of white blood cells, lymphocytes and platelets in the blood of diabetic rats, as illustrated in Supplementary Figures S7–S9. Notably, the administration of pCel led to significant improvements in the counts of white blood cells, lymphocytes, platelets and reticulocytes, detailed in Supplementary Figures S7–S10. Despite these improvements, circulating blood cells and other hematological parameters remained largely unchanged, as demonstrated in Supplementary Figures S10–S15.

**Figure 4.**
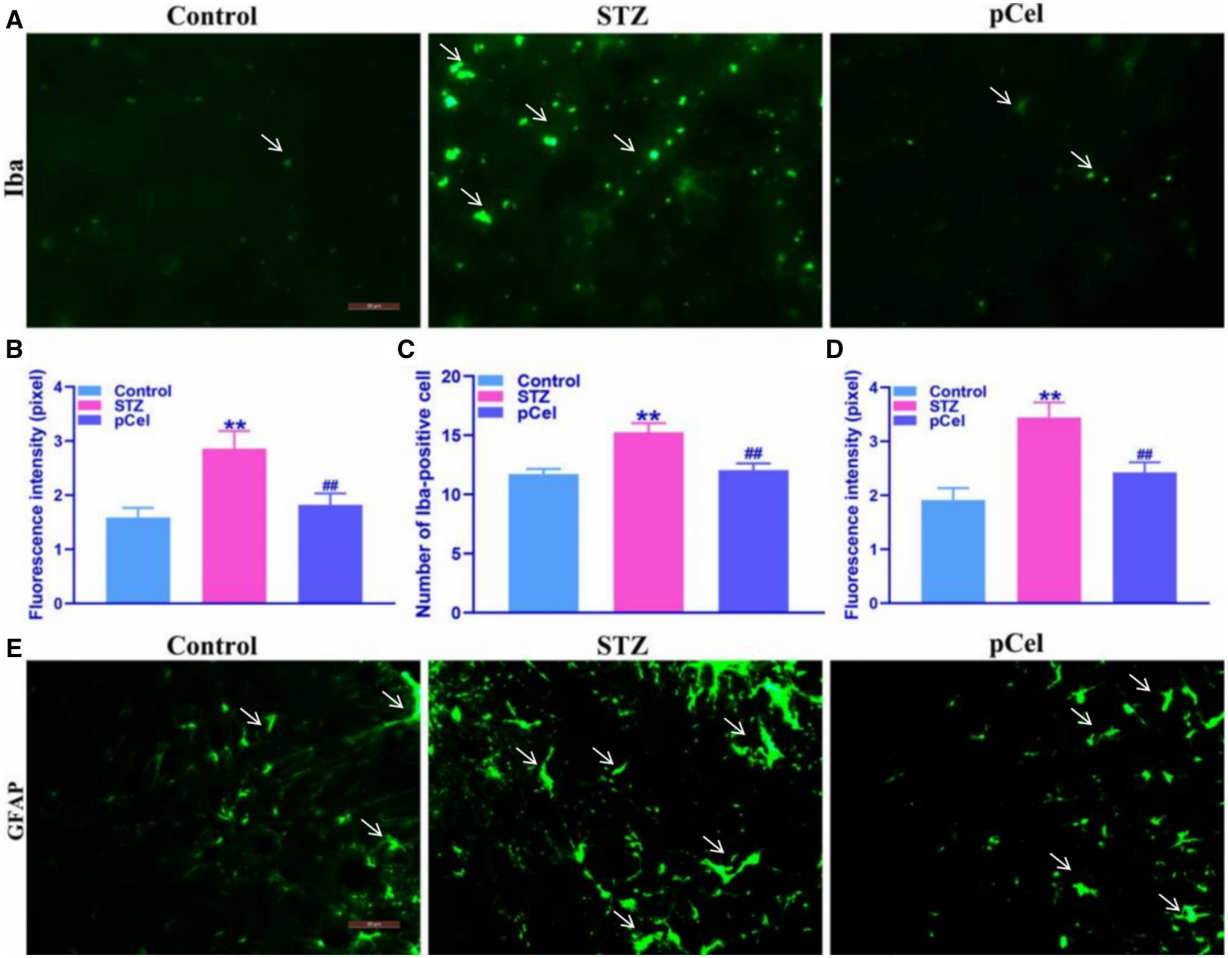
Inhibition of glia cell activation of pCel on Day 56. (**A**) Representative images of Iba-positive cells in STZ-induced diabetes (400×). (**B**, **C**) Representative quantification of Iba-positive fluorescence intensity and number in the sciatic nerve. (**D**) Representative quantification of GFAP-positive fluorescence intensity in the sciatic nerve. (**E**) Representative images of GFAP-positive cells in the sciatic nerve (400×). **P < 0.01, compared to the control group. ##*P* < 0.01, compared to the STZ group.

The role of glial cell activation in diabetic neuropathic pain has been previously identified [26]. In this context, STZ-treated animals exhibiting mechanical allodynia and elevated blood glucose levels were examined to assess the impact of pCel treatment on glial cell activation within the sciatic nerve. By Day 56, immunostaining demonstrated a marked upregulation in the expression of Iba1 (a microglial marker) and GFAP (an astrocyte marker) in the sciatic nerve, indicative of glial activation. Notably, the STZ-induced overexpression of both Iba1 and GFAP was significantly attenuated following pCel intervention, as shown in Figure 4A–D. These findings suggest that pCel not only effectively alleviates mechanical allodynia but also significantly suppresses glial cell activation in the sciatic nerve, potentially facilitating the restoration of the neural microenvironment.

### Decrease of gastrocnemius atrophy via pCel treatment *in vivo*

**Figure 5.**
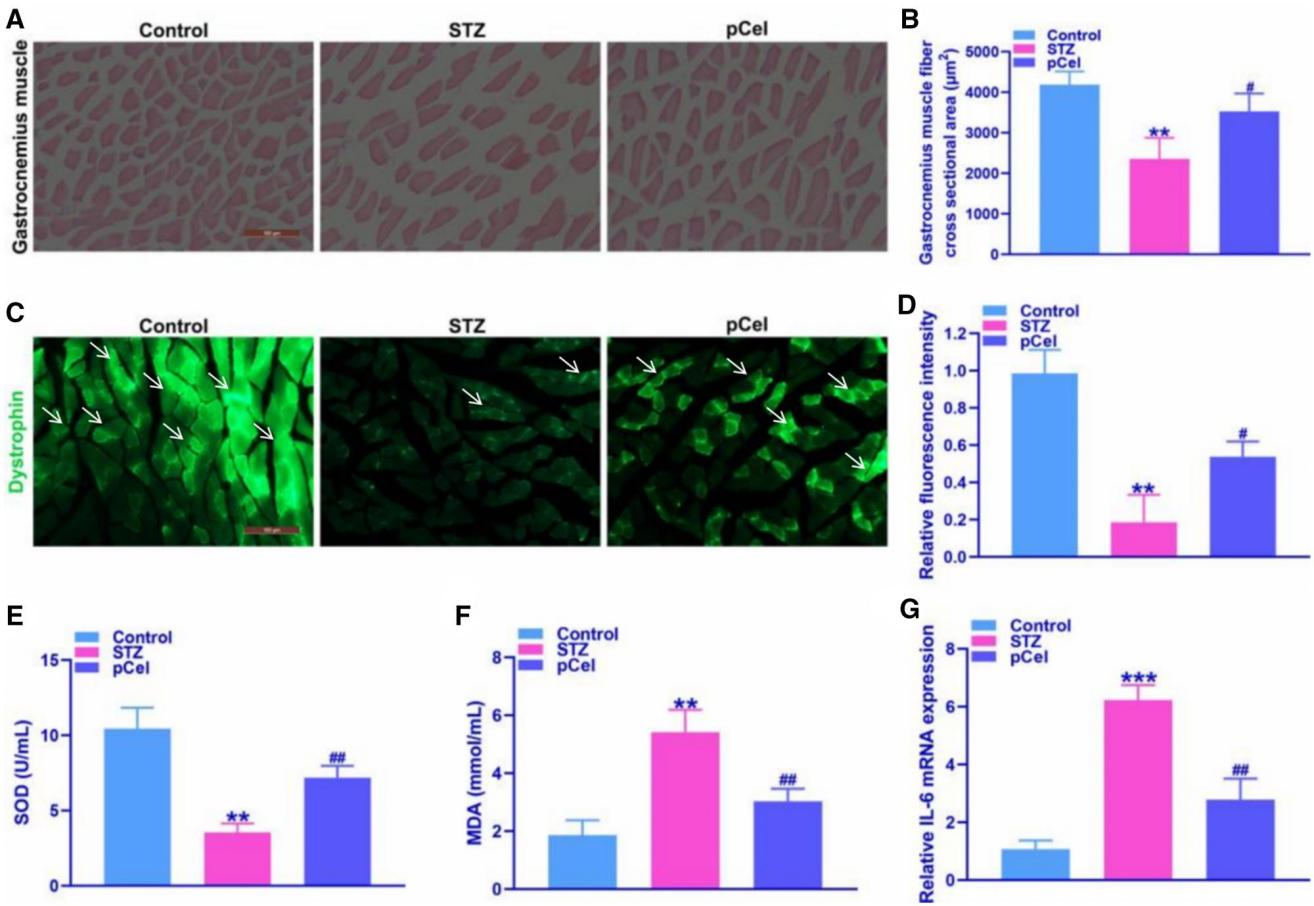
Decrease of gastrocnemius atrophy via pCel treatment *in vivo*. (**A**) Representative images of gastrocnemius muscle in STZ-induced diabetes (200×). (**B**) Representative quantification of gastrocnemius muscle via H&E staining. (**C**) Representative images of dystrophin-positive fluorescence intensity in the gastrocnemius muscle (200×). (**D**) Representative quantification of dystrophin-positive fluorescence intensity in the gastrocnemius muscle. (**E**, **F**) Representative quantification of SOD, MDA level in the gastrocnemius muscle. (**G**) Representative quantification of IL-6 mRNA in the gastrocnemius muscle. **P < 0.01, ***P < 0.001, compared to the control group. #*P* < 0.05, ##*P* < 0.01, compared to STZ group.

In clinical observations, an early symptom of diabetic neuropathy includes the weakening of small muscle reflexes in the feet [27]. The impact of pCel on the gastrocnemius muscle in diabetic rats was evaluated to assess its therapeutic efficacy (Figure 5). Results, as depicted in Figure 5A and B, indicate that 2 weeks after oral administration of pCel, the histopathological signs of muscular dystrophy, including necrotic myofibers, were substantially mitigated, aligning closely with observations in the control group.

Duchenne muscular dystrophy is characterized by the absence of dystrophin, a vital intracellular protein that ensures muscle cell membrane integrity by connecting the cell surface dystroglycan complex to the cytoskeleton [28]. In diabetic rats treated with pCel, immunofluorescence staining of gastrocnemius muscles showed a dystrophin-positive gain analogous to that observed in healthy rats, as evidenced in Figure 5C and D. This contrasts starkly with the pronounced dystrophin loss seen in untreated diabetic rats. Moreover, the levels of superoxide dismutase (SOD) and malondialdehyde (MDA), two critical indicators of ROS, were significantly better in the gastrocnemius muscles of STZ rats treated with pCel compared to those in the control group of diabetic rats (Figure 5E and F). Additionally, pCel treatment markedly reduced the STZ-induced upregulation of Interleukin-6 (IL-6) mRNA expression (Figure 5G), further underscoring pCel’s potent therapeutic effect in mitigating STZ-induced diabetic complications.

### Remyelination enhanced by pCel treatment *in vivo*

**Figure 6.**
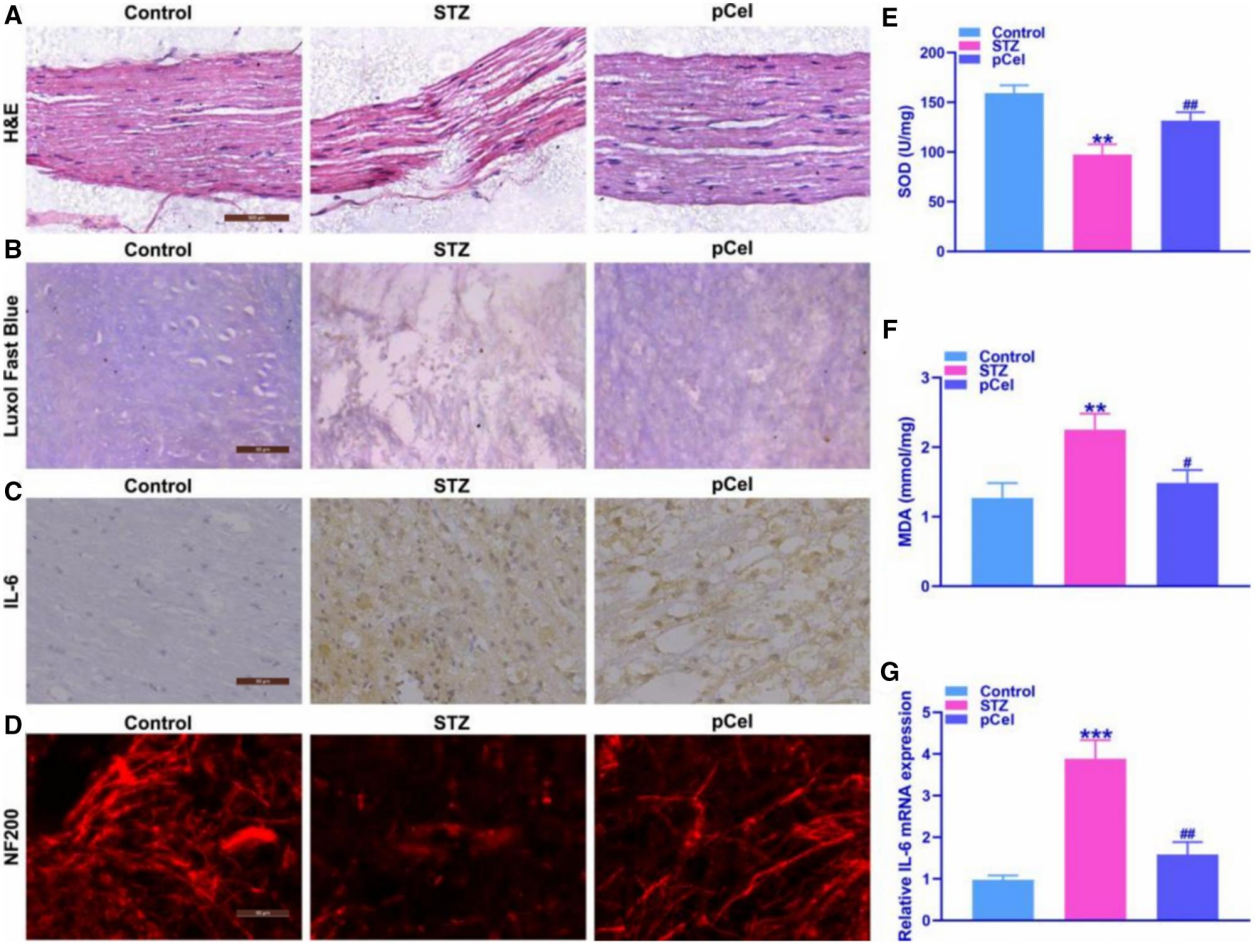
Remyelination enhanced by pCel treatment *in vivo*. (**A**) Representative images of the sciatic nerve in STZ-induced diabetes via H&E staining (100×). (**B**) Representative images of the sciatic nerve via luxol fast blue staining (400×). (**C**) Representative images of IL-6-positive fluorescence intensity in the sciatic nerve (400×). (**D**) Representative images of NF200-positive fluorescence intensity in the sciatic nerve (400×). (**E**, **F**) Representative quantification of SOD, MDA level in the sciatic nerve. (**G**) Representative quantification of IL-6 mRNA in the sciatic nerve. **P < 0.01, ***P < 0.001, compared to the control group. #*P* < 0.05, ##*P* < 0.01, compared to STZ group.

Myelin regeneration is a key process in achieving nerve regeneration. Studies have used three-dimensional printing of biomaterials to promote myelin regeneration in spinal cord injuries or peripheral nerve injuries [29, 30]. We further assessed the remyelination potential of pCel treatment, as shown in Figure 6. Histopathological analysis revealed that STZ-induced animals exhibited prominent axonal degeneration, myelinolysis and endometrial edema, compared to the control animals. Notably, pCel treatment ameliorated these pathological changes, improving axonal degeneration, myelinolysis and endometrial edema in rats (Figure 6A). Consequently, we delved into pCel’s capacity to foster axonal regeneration and mitigate the inflammatory response. The findings indicated that 2 weeks post-pCel administration in diabetic rats, the integrity of the myelin sheath closely resembled that of healthy counterparts (Figure 6B), and there was a significant suppression in the upregulation of IL-6 expression (Figure 6C). Additionally, sciatic nerve tissues marked with neurofilament 200 (NF200, red) showcased a considerable increase in NF200+ cells in the pCel-treated group compared to the STZ group (Figure 6D), further corroborating pCel’s effectiveness. The treatment with pCel in diabetic rats also resulted in notable improvements in ROS indicators and the levels of proinflammatory cytokines, underscoring the therapeutic efficacy of pCel in addressing complications arising from diabetic neuropathy.

Diabetic peripheral neuropathy is one of the diseases increasingly threatening human health with the aging population and rising incidence of diabetes. Its treatment primarily involves controlling blood glucose levels and alleviating local symptoms such as neuropathic pain. Current research mainly focuses on drug therapies and the investigation of molecular mechanisms to find effective treatments. Regarding the molecular mechanisms of onset, researchers have explored several metabolic pathways in diabetic peripheral neuropathy, including the polyol pathway, hexosamine biosynthesis pathway, protein kinase C pathway and the excessive production of advanced glycation end-products [31–33]. ROS damage and mitochondrial dysfunction are likely the ultimate pathways [34].

In recent years, many researchers have focused on engineered biomaterial treatments for diabetic peripheral nerve injury. In 2021, Anamika *et al.* [35] proposed using exosomes derived from bone marrow mesenchymal stem cells combined with electrical stimulation to treat diabetic peripheral neuropathy. In electrophysiological studies, exosomes significantly enhanced electrophysiological parameters such as motor nerve conduction velocity and compound muscle action potential. At the cellular/molecular level, exosomes again showed a notable impact on histopathology, although this effect could not be enhanced by additional electrical stimulation. Their engineered exosomes improved nerve conduction function in peripheral nerve injuries to some extent, but better effects were achieved when combined with electrical stimulation treatment. In 2023, Lin *et al.* [36] proposed using tetrahedral framework nucleic acids loaded with resveratrol (RSV) as ‘nano-guardians’ of mitochondria for treating diabetic peripheral neuropathy. Through tail vein injection, the bioavailability of RSV was improved, mainly functioning through redox regulation and energy metabolism, providing a new approach for the treatment of diabetic peripheral neuropathy.

In this study, we developed biodegradable polylactic acid glycolic acid nanoparticles obtained by loading triptolide (pCel) to improve somatic cell neuropathic pain caused by diabetes. The pCel was designed to be administered orally, which could be more convenient for potential clinical use. With the treatment of pCel, ROS and apoptosis levels were remarkably decreased *in vitro*. The progress of streptozotocin (STZ)-induced diabetes is shown by the upregulation of renal function indexes (blood urea nitrogen, creatinine), liver function indexes (bilirubin, alkaline phosphatase) and the downregulation of albumin and globulin, while pCel administration reversed these aberrancies. Moreover, oral pCel treatment significantly prevented mechanical allodynia and the activation of the sciatic glial cells in diabetes rats and it also promotes myelin regeneration. These results suggest that with good stability, biocompatibility and high catalytic activity, this artificial biodegradable nanomedicine will be an effective and exciting perspective toward the application of chronic painful conditions with diabetic neuropathy.

However, this study still has some limitations. We focused on the stability, biocompatibility and catalytic activity of pCel, which are essential considerations for its clinical translation. However, providing data or discussion on potential off-target effects, systemic toxicity or long-term safety profiles of pCel would be valuable for assessing its overall safety profile and potential clinical applications, and more in-depth studies on the signaling pathways or targets of action need to be addressed in our future research.

## Conclusion

The findings from this investigation highlight the neuroprotective capacity of orally administered pCel in safeguarding neurons against H_2_O_2_-induced excitotoxicity, facilitating the repair of damaged sciatic nerves, and consequently, ameliorating somatic neuropathic pain. pCel administration notably reduces the expression of ROS, apoptosis and inflammation both *in vitro* and *in vivo*. Histological improvements in the gastrocnemius muscle and sciatic nerve tissue, alongside the alleviation of somatic neuropathic pain in diabetic models, were promptly observed following pCel treatment. The efficacy of pCel suggests that oral delivery of such nanomaterials holds significant promise for advancing treatment modalities for nerve injuries beyond just diabetic neuropathy. Future research endeavors will focus on incorporating therapeutics into pCel to enhance its pain-relieving properties. In essence, our study offers promising insights into employing neuroprotective pCel formulations for managing severe neuropathic pain conditions.

## Supplementary Material

rbae087_Supplementary_Data

## Data Availability

The datasets used or analyzed during the current study are available on reasonable request.
