# Supplementary material for: Biodegradable microspheres via orally deliver celastrol with ameliorated neuropathic pain in diabetes rats
Source: Regen Biomater. 2024 Jul 17;11:rbae087. doi: 10.1093/rb/rbae087 (PMC11272178; doi:10.1093/rb/rbae087)
Supplement: rbae087_Supplementary_Data [file rbae087_supplementary_data.docx]

Supplementary material

**Biodegradable spheres via orally deliver celastrol with ameliorated somatic neuropathic pain induced by diabetes**

Haosen Zhao^1,#^, Shurui Chen^2,#^, Sen Lin^1,*^, Xifan Mei^1,*^,

^1^Department of Orthopaedic Rehabilitation, Third Affiliated Hospital of Jinzhou Medical University, Jinzhou, P. R. China.

^2^Cardiac Intensive Care Unit, Cardiovascular Hospital, Guangdong Second Provincial General Hospital, Guangzhou, P. R. China.

# These authors contributed equally to this work and should be considered as co-first authors.

**^*^Corresponding Author**

Sen Lin, aldrin_lin@163.com

Xifan Mei, [meixifan@jzmu.edu.cn](mailto:meixifan@jzmu.edu.cn)

**This file includes:** Figure S1 To Figure S15.


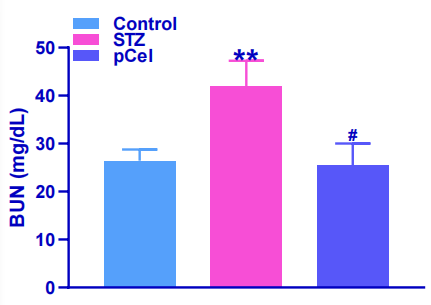


**Supplementary Figure 1.** Representative quantification of of BUN.


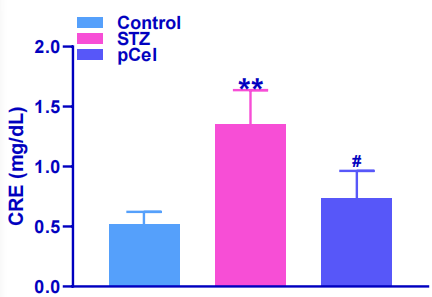


**Supplementary Figure 2.** Representative quantification of of CRE.


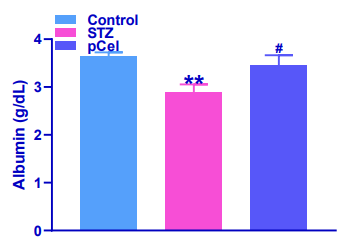


**Supplementary Figure 3.** Representative quantification of of albumin.


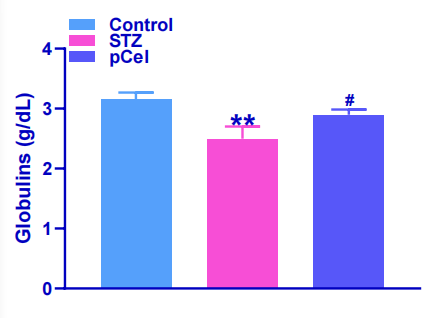


**Supplementary Figure 4.** Representative quantification of of globulin.


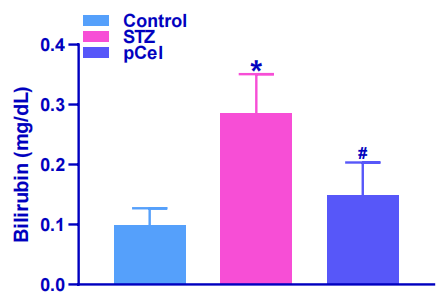


**Supplementary Figure 5.** Representative quantification of of bilirubin.


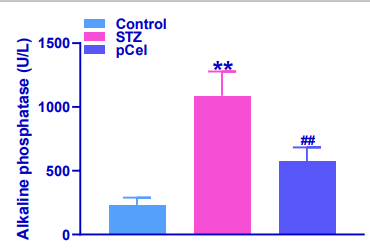


**Supplementary Figure 6.** Representative quantification of of alkaline phosphatase.


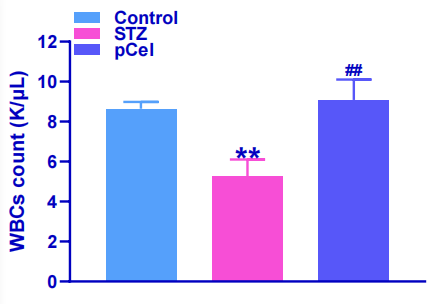


**Supplementary Figure 7.** Representative quantification of of WBCs.


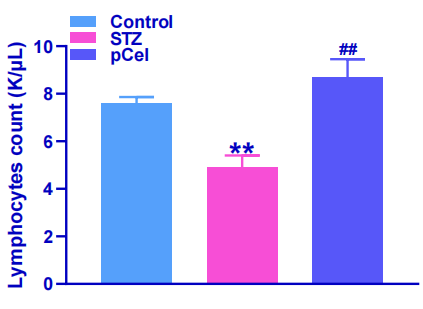


**Supplementary Figure 8.** Representative quantification of of lymphocytes.


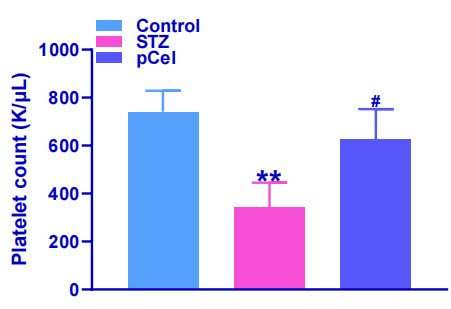


**Supplementary Figure 9.** Representative quantification of of platelets.


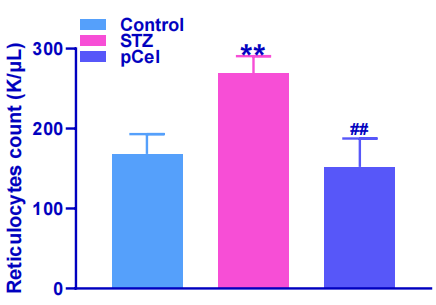


**Supplementary Figure 10.** Representative quantification of of reticulocytes.


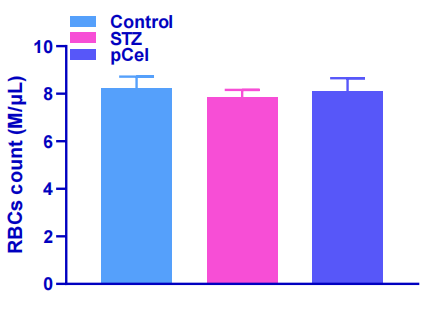


**Supplementary Figure 11.** Representative quantification of of RBCs.


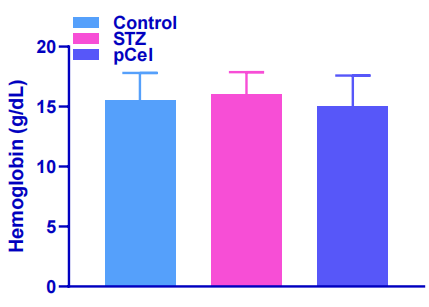


**Supplementary Figure 12.** Representative quantification of of hemohlobin.


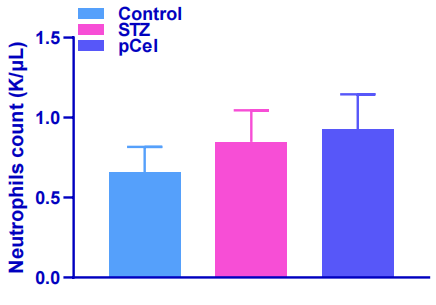


**Supplementary Figure 13.** Representative quantification of of neutrophils.


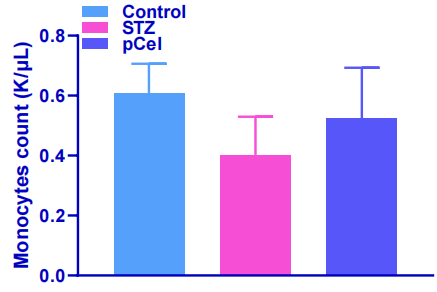


**Supplementary Figure 14.** Representative quantification of of monocytes.


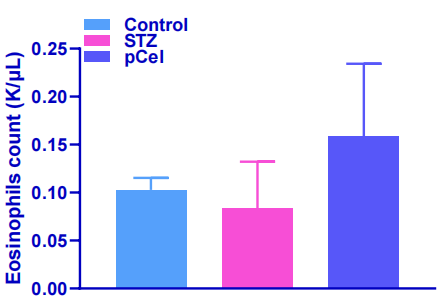


**Supplementary Figure 15.** Representative quantification of of eosinophils.
